# Impact of Alexithymia on the Lipid Profile in Major Depressed Individuals

**DOI:** 10.1155/2022/5450814

**Published:** 2022-06-16

**Authors:** Camille Point, Benjamin Wacquier, Marjorie Dosogne, Mohammed Al Faker, Hadrien Willame, Gwenolé Loas, Matthieu Hein

**Affiliations:** Erasme Hospital, Department of Psychiatry and Sleep Laboratory, Université Libre de Bruxelles (ULB), Brussels, Belgium

## Abstract

**Background:**

The cooccurrence of major depression and dyslipidaemia is associated with negative cardiovascular outcome, which seems to justify a better identification of the factors favouring the development of dyslipidaemia in major depressed individuals. In the literature, there are arguments in favour of a special relationship between dyslipidaemia and alexithymia. However, despite a high prevalence of alexithymia in major depressed individuals, no study has investigated the impact of this personality trait on the lipid profile in this particular subpopulation. Given these elements, the aim of this study was therefore to investigate the risk of dyslipidaemia associated with alexithymia in major depressed individuals to allow better cardiovascular prevention in this subpopulation. *Subjects and Methods*. Demographic and polysomnographic data from 242 major depressed individuals recruited from the clinical database of the sleep laboratory were analysed. Only individuals with a diagnosis of dyslipidaemia according to the diagnostic criteria of the *International Diabetes Federation* at admission were included in the “dyslipidaemia” group. Logistic regression analyses were used to determine the risk of dyslipidaemia associated with alexithymia in major depressed individuals.

**Results:**

The prevalence of dyslipidaemia was 43.8% in our sample of major depressed individuals. After adjusting for the main confounding factors, multivariate logistic regression analyses demonstrated that alexithymia was a risk factor for dyslipidaemia in major depressed individuals.

**Conclusions:**

In this study, we found that alexithymia is a risk factor for dyslipidaemia in major depressed individuals, which seems to justify better identification and adequate management of this personality trait in order to allow a better lipid profile in this subpopulation at high cardiovascular risk.

## 1. Introduction

Psychopathologically, alexithymia is characterised by several interconnected elements: (1) difficulties identifying feelings and emotions, (2) limited ability to communicate feelings and emotions to others, (3) problems distinguishing between emotions and bodily sensations that relate to these emotions, (4) logical and rigid thinking style that does not account for emotions (externally oriented thinking style), and (5) restricted imaginal processes [1]. Along with this negative effect on psychic functioning, alexithymia also seems to have a deleterious cardiometabolic impact favouring the occurrence of some components of the metabolic syndrome [2]. In the literature, there are notably many arguments in favour of a special interplay between alexithymia and dyslipidaemia [3]. Although the specific pathophysiology of this particular relationship is not yet fully understood, the existence of some deleterious lifestyle habits (sedentary lifestyle, higher alcohol consumption, reduced physical activity, and inadequate diet habits) in alexithymic individuals [4] could play a central role in the development of dyslipidaemia given their negative impact on lipid metabolism [5]. In addition, the prevalence of dyslipidaemia appears to be higher in alexithymic individuals than in nonalexithymic individuals [6], which seems to indicate that this personality trait could be a specific risk factor for dyslipidaemia [7]. Moreover, in some subpopulations, these alterations in lipid metabolism related to alexithymia could play a major role in the development of atherosclerotic plaques that are associated with a less favourable cardiovascular prognosis [8, 9]. Given these elements, it seems necessary to conduct additional investigations in subpopulations at high risk of alexithymia to allow a better understanding of the potential implication of this personality trait in the occurrence of dyslipidaemia.

Similar to other psychiatric conditions (panic disorder, obsessive-compulsive disorder, and suicidal behaviours), several elements seem to suggest that there is a special relationship between major depression and alexithymia [10–12]. Indeed, in major depressed individuals, the prevalence of alexithymia is higher than that in the general population [13]. In addition, major depression has been shown to be a risk factor for alexithymia [14]. On the other hand, alexithymia appears to be associated with altered lipid profiles in some psychiatric disorders (obsessive-compulsive disorder) [12]. Nevertheless, despite this potential negative impact of this personality trait on the lipid profile in some psychiatric populations and its high prevalence in major depression, few studies have so far investigated the risk of dyslipidaemia associated with alexithymia in major depressed individuals [15]. However, alexithymia could play a major role in the pathophysiology of deleterious cardiovascular complications associated with the cooccurrence of major depression and dyslipidaemia [16]. Thus, it could be interesting to study the risk of dyslipidaemia associated with alexithymia in major depressed individuals in order to allow the establishment of more targeted therapeutic strategies in this subpopulation at high cardiovascular risk [17].

The objective of this study was therefore to empirically investigate the risk of dyslipidaemia associated with alexithymia in a large sample of major depressed individuals. The aim of this approach was to provide healthcare professionals caring for major depressed individuals with reliable data regarding the risk of dyslipidaemia associated with this personality trait in order to allow better management of this cardiovascular risk factor in this particular subpopulation.

## 2. Material and Method

The methodology used in this study is similar to that used in previous studies of our research group [18].

### 2.1. Population

**Figure 1:**
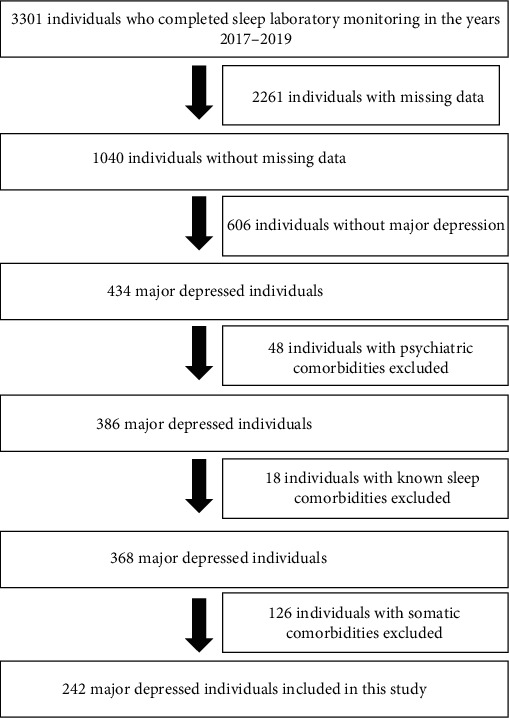
Selection diagram of major depressed individuals included in this study.

242 major depressed individuals were recruited consecutively from the clinical database of the Erasme Hospital Sleep Laboratory, which contains the data of 3301 individuals who performed a sleep recording between 2017 and 2019 (Figure 1). In our study, we did not recruit individuals without major depression because our objective was to focus on the subpopulation of major depressed individuals where the cooccurrence of dyslipidaemia may have a deleterious impact on cardiovascular outcome [16].

These major depressed individuals were referred to the sleep laboratory by physicians specialised in sleep medicine after an outpatient consultation during which a preliminary assessment of their complaints related to sleep, their ongoing psychotropic/somatic treatments, and their somatic/psychiatric comorbidities was systematically carried out in order to allow a first diagnostic hypothesis. These polysomnographic recordings were performed in these major depressed individuals to allow an objective assessment of their sleep complaints and exclude the presence of comorbid sleep disorders negatively impacting mood regulation.

The inclusion criteria were age ≥ 18 years and the presence of a major depressive episode according to the DSM-5 diagnostic criteria [19].

The exclusion criteria were the presence of psychiatric disorders other than major depression (including intellectual disability), the presence of severe uncontrolled somatic pathologies (chronic liver pathologies, chronic pancreatic pathologies, chronic pulmonary pathologies, severe cardiovascular pathologies, severe renal pathologies, autoimmune pathologies, severe endocrine pathologies, severe neurological pathologies, and pathologies altering the activity of the hypothalamic-pituitary-adrenal axis such as Cushing's syndrome), the presence of inflammatory or infectious diseases, the presence or history of head trauma, the presence or history of central nervous system damage that may affect the respiratory centres, the presence of craniofacial or thoracic malformations, the presence of ongoing pregnancy, the presence of obstructive sleep apnoea syndrome being treated before the sleep laboratory, the presence of predominantly central sleep apnoea syndrome, the presence of central hypersomnia, the presence of parasomnia, and the presence or history of drug addiction.

### 2.2. Medical and Psychiatric Assessment of Participants

During their admission to the Erasme Hospital Sleep Laboratory, major depressed individuals included in this study benefited from a review of their medical records and a complete somatic assessment (including blood test, electrocardiogram, day electroencephalogram, and urinalysis) in order to allow a systematic diagnosis of their potential somatic pathologies.

Following this somatic assessment, dyslipidaemia was defined as present if one of the following criteria were present:

- Self-reported diagnosis of dyslipidaemia biologically demonstrated according to the diagnostic criteria of the *International Diabetes Federation* [20]
- Or taking medication for dyslipidaemia or plasma triglyceride levels ≥ 150 mg/dL or plasma HDL-cholesterol levels < 40 mg/dL for men or plasma HDL-cholesterol levels < 50 mg/dL for women [20]

Subsequently, a systematic psychiatric assessment based on the DSM-5 diagnostic criteria [19] was performed by a unit psychiatrist in major depressed individuals recruited for this study to confirm the diagnoses of major depressive episodes highlighted during the outpatient assessment and to exclude the presence of comorbid psychiatric disorders (including intellectual disability).

Finally, a series of self-questionnaires were completed by major depressed individuals included in this study to assess the severity of their subjective complaints of depression (Beck Depression Inventory [BDI-II]), daytime sleepiness (Epworth Sleepiness Scale), and insomnia (Insomnia Severity Index) (detailed description available in Supplementary Data—Annex 1). Regarding alexithymia, this personality trait was investigated by the Toronto Alexithymia Scale (TAS-20) [21]. Each item of this scale may be scored from “strongly agree” to “strongly disagree” on a five-point Likert scale. Three different levels of alexithymia are considered: nonalexithymic (scores less than 51), moderately alexithymic (scores between 51 and 60), and severely alexithymic (scores over 60). Based on this scale, alexithymia was considered to be present when the score was >50 in major depressed individuals recruited for this study [21].

### 2.3. Sleep Assessment of Participants

In major depressed individuals included in this study, a specific sleep interview was performed by a unit psychiatrist during their admission to the sleep laboratory in order to systematically investigate their complaints related to sleep including sleeping habits, severity of self-reported insomnia complaints (difficulty falling asleep, repeated nighttime awakenings, early morning awakening, and nonrestorative sleep), symptoms of sleep apnoeas (snoring and self-reported apnoeas), symptoms of restless leg syndrome (impatience of legs with or without abnormal sensations: aggravated by rest, partially or temporarily relieved by movements, and increased during evening or night), and abnormal nocturnal movements (such as periodic limb movements during sleep).

During their stay in the sleep laboratory, major depressed individuals included in this study benefited from a polysomnographic recording from which the data were collected for analysis. The patients went to bed between 22:00 and 24:00 and got up between 6:00 and 800, following their usual schedule. During bedtime hours, the subjects were recumbent and the lights were turned off. Daytime naps were not permitted.

The polysomnographic recordings performed in our unit meet the recommendations of the *American Academy of Sleep Medicine* [22]. The description of the applied polysomnography montage is available in Supplementary Data—Annex 2. Polysomnographic recordings were visually scored by specialised technicians according to the criteria of the *American Academy of Sleep Medicine* [23]. The description of scoring criteria and diagnostic criteria used for the diagnosis of sleep disorders is available in Supplementary Data—Annex 3.

Thanks to these different steps, a systematic diagnosis of potential comorbid sleep disorders was performed in major depressed individuals recruited for this study.

### 2.4. Statistical Analyses

Statistical analyses were performed using Stata 14. The normal distribution of the data was verified using histograms, boxplots, and quantile-quantile plots whereas the equality of variances was checked using the Levene test.

In order to allow our analyses, we divided our sample of major depressed individuals into a control group without dyslipidaemia and a patient group with dyslipidaemia. Only major depressed individuals with a diagnosis of dyslipidaemia according to the diagnostic criteria of the *International Diabetes Federation* were included in the “dyslipidaemia” group [20].

Categorical data were described by percentages and numbers whereas continuous variables were described by their median and P25-P75. Since most continuous data followed an asymmetric distribution, we decided to use nonparametric tests for all these variables (Wilcoxon test) in order to highlight significant differences between the medians (P25-P75) observed in the different groups of major depressed individuals. Finally, the categorical data were described by percentage and were analysed with chi^2^ tests.

Univariate logistic regression models were used to study the risk of dyslipidaemia associated with alexithymia and the potential confounding factors (detailed description available in Supplementary Data—Annex 4). In multivariate logistic regression models, the risk of dyslipidaemia associated with alexithymia was only adjusted for significant confounding factors during univariate analysis. These different confounding factors were introduced hierarchically in the different multivariate logistic regression models.

The adequacy of the final model was verified by the Hosmer and Lemeshow test whereas the specificity of the model was verified by the Link test.

The results were considered significant when the *P* value was <0.05.

## 3. Results

### 3.1. Polysomnographic Data

Compared to major depressed individuals without dyslipidaemia, major depressed individuals with dyslipidaemia showed an increase in stage 1, microarousal index, obstructive apnoea-hypopnoea index, oxygen desaturation index, total time under 90% of SaO_2_, and periodic limb movement's index (Table 1). There were no significant differences between the two groups for the other polysomnographic parameters (Table 1).

### 3.2. Demographic Data

In major depressed individuals with dyslipidaemia, body mass index, triglyceride levels, and CRP levels were higher than those in major depressed individuals without dyslipidaemia (Table 2). Furthermore, major depressed individuals with dyslipidaemia had lower HDL-cholesterol levels than major depressed individuals without dyslipidaemia (Table 2). Finally, overweight, obesity, snoring, obstructive sleep apnoea syndrome, hypertension, type 2 diabetes, CRP levels ≥ 1 and <3 mg/L, CRP levels ≥ 3 mg/L, and alexithymia were more frequent in major depressed individuals with dyslipidaemia than in major depressed individuals without dyslipidaemia (Table 2). The two groups did not differ significantly for other demographic parameters (Table 2).

### 3.3. Prevalence of Dyslipidaemia in Major Depressed Individuals

The prevalence of dyslipidaemia was 43.8% (*n* = 106) in our sample of major depressed individuals (Table 2).

### 3.4. Univariate Regression Analyses

Body mass index ≥ 25 and <30 kg/m^2^, body mass index ≥ 30 kg/m^2^, snoring, obstructive apnoea syndrome sleep, hypertension, type 2 diabetes, CRP levels ≥ 1 and <3 mg/L, CRP levels ≥ 3 mg/L, and alexithymia were associated with an increased risk of dyslipidaemia in major depressed individuals (Table 3).

### 3.5. Multivariate Regression Analyses

After adjusting for the main significant confounders in univariate analyses, multivariate logistic regression analyses demonstrated that alexithymia was a risk factor for dyslipidaemia in major depressed individuals (Table 4).

### 3.6. Lipid Profile according to Alexithymic Status

Major depressed individuals with alexithymia had higher levels of total cholesterol and triglycerides than major depressed individuals without alexithymia. There were no significant differences between the two groups for HDL-cholesterol levels (Table 5).

## 4. Discussion

The prevalence of dyslipidaemia demonstrated in our study (43.8%) seems to be lower than that of the study by Seo and Je (49.7%) [24], which could be explained by differences in the populations recruited. Indeed, in this study, the prevalence of diabetes disorders in major depressed individuals was higher than that in our study. However, diabetes disorders are major risk factors for dyslipidaemia [25], which may have led to an overestimation of the prevalence of dyslipidaemia in the study by Seo and Je [24]. Furthermore, the prevalence of dyslipidaemia in our study appears to be higher than that of the study by Hidese et al. (14.6%) [26], which could be explained by a better cardiometabolic profile in major depressed individuals included in this study (lower body mass index–lower prevalence of hypertension and diabetes disorders). However, the presence of this better cardiometabolic profile may have favoured an underestimation of the prevalence of dyslipidaemia in the study by Hidese et al. [26] given the central role played by these different cardiometabolic factors in the pathophysiology of dyslipidaemia [27]. Finally, the prevalence of dyslipidaemia demonstrated in our study seems to be consistent with that of studies by Hein et al. (41.1%) and Park and Lee (42.4%) that had included populations of major depressed individuals similar to the current study [28–30]. Thus, regardless of some methodological differences, we confirmed that dyslipidaemia is a frequent comorbidity in major depressed individuals, which seems to justify a more systematic screening of this problem by healthcare professionals caring for major depressed individuals in order to allow better management of this cardiovascular risk factor in this particular subpopulation.

Similar to the literature, we have confirmed that alexithymia is a frequent personality trait in major depressed individuals [31]. In addition, we have demonstrated an increased risk of dyslipidaemia associated with alexithymia in major depressed individuals, which seems to be consistent with the data available for some subpopulations [2, 7, 32]. Physiopathologically, several elements could help to better understand this particular relationship between alexithymia and dyslipidaemia in major depression. First, in major depressed individuals, alexithymia may be associated with the occurrence of major psychological distress leading to the development of avoidance strategies [33, 34]. However, some of these strategies for avoiding the psychic pain associated with alexithymia are characterised by the development of inadequate eating behaviours (such as “binge eating”) favouring the occurrence of obesity that plays a central role in the pathophysiology of the different components of the metabolic syndrome (such as dyslipidaemia) [33, 34]. Second, in major depressed individuals, preexisting alterations in some adipocytokines (such as adiponectin) involved in the regulation of lipid metabolism [35] could be potentiated by alexithymia given its negative impact on the secretion of these hormones [36]. However, the presence of lower peripheral adiponectin levels could promote the development of dyslipidaemia through several deleterious mechanisms (obesity, chronic inflammation, insulin resistance, blood pressure deregulation, and changes in the lipid profile) [37]. Third, given its potential direct negative effect on blood pressure regulation and glycaemic metabolism [38, 39], alexithymia could contribute to the less favourable cardiometabolic profile (uncontrolled hypertension and diabetes) present in some major depressed individuals [40, 41]. However, these two deleterious cardiometabolic conditions are frequently associated with alterations in lipid metabolism promoting the occurrence of dyslipidaemia [42]. Thus, following these various elements, it seems necessary to identify and manage complaints of alexithymia in major depressed individuals in order to allow a better lipid profile in this subpopulation at high cardiovascular risk.

The demonstration in our study of an increased risk of dyslipidaemia associated with alexithymia could open up new therapeutic perspectives in major depressed individuals with dyslipidaemia. Indeed, after adequate treatment of dyslipidaemia [43], the establishment of therapeutic strategies targeted on alexithymia seems to be necessary in major depressed individuals with dyslipidaemia in order to avoid the maintenance of lifestyle habits deleterious for the lipid metabolism related to this personality trait (sedentary lifestyle, higher alcohol consumption, reduced physical activity, and inadequate diet habits) [44, 45]. Among the therapeutic strategies available, there seem to be arguments in favour of the effectiveness of some psychotherapeutic treatments on complaints of alexithymia although there is currently no standardised treatment for this personality trait [46, 47]. However, in case of implementation of these therapeutic strategies in major depressed individuals with alexithymia, it will be important to take into account the potential negative impact of this personality trait on the therapeutic alliance in order to potentiate the chances of success of these psychotherapeutic treatments [48]. Finally, alongside these potential more targeted treatments for alexithymia, the establishment of optimal treatment for major depression is essential since complaints of alexithymia may be induced or aggravated by major depressive episodes [49, 50].

### 4.1. Limitations

The results obtained in our study come from retrospective data that, even if they have been encoded in a systematic manner, cannot be verified directly with the subject in most cases, which means that our results need to be replicated in prospective studies. Furthermore, we only focused on dyslipidaemia, which means that our results cannot be generalised to other cardiovascular risk factors (such as diabetes disorders or hypertension). In addition, since psychiatric disorders other than major depression were exclusion criteria in our study, our results can only be applied to major depression, which may possibly limit their interpretation. Finally, our database only contains major depressed individuals who have agreed to perform a sleep laboratory, which may also limit the generalisation of our results.

## 5. Conclusion

We demonstrated that dyslipidaemia was a frequent pathology in our sample of major depressed individuals. In addition, we have shown that alexithymia is a risk factor for dyslipidaemia in major depressed individuals, which seems to justify better identification and adequate management of this personality trait in order to allow a better lipid profile in this subpopulation at high cardiovascular risk.

## Figures and Tables

**Table 1:** Polysomnographic data (*n* = 242).

	Whole sample (*n* = 242)	Depression without dyslipidaemia (*n* = 136)	Depression with dyslipidaemia (*n* = 106)	*P* value
Sleep latency (min)	59.5 (35.0–99.0)	61.5 (38.5–94.5)	56.0 (33.0–101.0)	0.468
Sleep efficiency (%)	75.5 (65.0–83.0)	75.5 (66.0–83.0)	75.5 (65.0–83.0)	0.999
Sleep period time (min)	440.5 (410.0–466.0)	438.5 (410.0–464.5)	442.5 (408.0–470.0)	0.538
Total sleep time (min)	391.5 (335.0–428.0)	389.5 (334.5–425.5)	396.0 (335.0–430.0)	0.869
% stage 1	7.0 (5.0–9.0)	6.0 (4.0–8.0)	7.0 (5.0–11.0)	0.002
% stage 2	50.0 (44.0–58.0)	50.0 (44.0–58.0)	51.0 (45.0–58.0)	0.898
% slow-wave sleep	11.0 (5.0–17.0)	11.0 (5.0–19.0)	9.0 (4.0–15.0)	0.090
% REM sleep	17.0 (12.0–21.0)	17.0 (12.0–21.0)	17.0 (12.0–21.0)	0.626
REM latency (min)	82.0 (66.0–139.0)	83.5 (66.0–136.0)	80.5 (65.0–152.0)	0.903
% wake after sleep onset	8.0 (5.0–16.0)	8.0 (5.0–16.0)	9.0 (5.0–17.0)	0.197
Number of awakenings	22 (17–30)	22 (16–29)	24 (18–32)	0.052
Microarousal index	10 (6–15)	9 (6–14)	11 (6–19)	0.038
Apnoea-hypopnoea index	2.8 (0.6–12.8)	1.7 (0.4–8.5)	5.7 (1.0–16.7)	0.001
Oxygen desaturation index	1 (0–4)	1 (0–3)	2 (0–9)	0.001
Total time under 90% of SaO_2_ (min)	0.0 (0.0–5.0)	0.0 (0.0–1.0)	0.5 (0.0–14.0)	0.001
PLMs index	4.0 (0.7–10.7)	2.9 (0.1–9.0)	6.0 (1.4–15.0)	0.008
	Median (P25-P75)	Median (P25-P75)	Median (P25-P75)	Wilcoxon test

PLMs = periodic limb movements during sleep; REM = rapid eye movement.

**(a):** 

Variables	Categories	%	Depression without dyslipidaemia	Depression with dyslipidaemia	*P* value chi^2^
Gender	Female (*n* = 139)	57.4%	61.0%	52.8%	0.201
	Male (*n* = 103)	42.6%	39.0%	47.2%	
BMI (kg/m^2^)	≥18 & <25 (*n* = 75)	31.0%	41.2%	17.9%	<0.001
	≥25 & <30 (*n* = 87)	36.0%	31.6%	41.5%	
	≥30 (*n* = 80)	33.0%	27.2%	40.6%	
Age (years)	<50 (*n* = 159)	65.7%	69.1%	61.3%	0.205
	≥50 (*n* = 83)	34.3%	30.9%	38.7%	
Benzodiazepine receptor agonists	No (*n* = 196)	81.0%	83.1%	78.3%	0.346
	Yes (*n* = 46)	19.0%	16.9%	21.7%	
Antidepressant therapy	No (*n* = 162)	66.9%	66.2%	67.9%	0.774
	Yes (*n* = 80)	33.1%	33.8%	32.1%	
Other psychotropic treatments	No (*n* = 217)	89.7%	90.4%	88.7%	0.655
	Yes (*n* = 25)	10.3%	9.6%	11.3%	
Smoking	No (*n* = 192)	79.3%	83.1%	74.5%	0.103
	Yes (*n* = 50)	20.7%	16.9%	25.5%	
Alcohol	No (*n* = 131)	54.1%	52.9%	55.7%	0.913
	Occasional (*n* = 87)	36.0%	36.8%	34.9%	
	Regular (*n* = 24)	9.9%	10.3%	9.4%	
Caffeine	No (*n* = 33)	13.6%	15.4%	11.3%	0.354
	Yes (*n* = 209)	86.4%	84.6%	88.7%	
Snoring	No (*n* = 87)	36.0%	44.9%	24.5%	0.001
	Yes (*n* = 155)	64.0%	55.1%	75.5%	
OSAS	No (*n* = 141)	58.3%	67.7%	46.2%	0.001
	Yes (*n* = 101)	41.7%	32.3%	53.8%	
Insomnia disorders	No (*n* = 45)	18.6%	16.9%	20.8%	0.651
	Sleep deprivation alone (*n* = 27)	11.2%	10.3%	12.3%	
	With sleep duration ≥ 6 hours (*n* = 123)	50.8%	54.4%	46.2%	
	With sleep duration < 6 hours (*n* = 47)	19.4%	18.4%	20.7%	
Sleep movement disorders	None (*n* = 183)	75.6%	77.9%	72.6%	0.630
	Moderate to severe PLMs alone (*n* = 31)	12.8%	11.8%	14.2%	
	RLS alone or combined with PLMs (*n* = 28)	11.6%	10.3%	13.2%	
Excessive daytime sleepiness	No (*n* = 111)	45.9%	49.3%	41.5%	0.230
	Yes (*n* = 131)	54.1%	50.7%	58.5%	
Depression severity	Mild (*n* = 78)	32.2%	36.8%	26.4%	0.108
	Moderate (*n* = 110)	45.5%	39.7%	52.8%	
	Severe (*n* = 54)	22.3%	23.5%	20.8%	
Hypertension	No (*n* = 150)	62.0%	70.6%	50.9%	0.002
	Yes (*n* = 92)	38.0%	29.4%	49.1%	
Type 2 diabetes	No (*n* = 211)	87.2%	96.3%	75.5%	<0.001
	Yes (*n* = 31)	12.8%	3.7%	24.5%	
CRP (mg/L)	<1 (*n* = 82)	33.9%	41.9%	23.6%	0.010
	≥1 & <3 (*n* = 80)	33.1%	27.9%	39.6%	
	≥3 (*n* = 80)	33.0%	30.2%	36.8%	
Alexithymia	No (*n* = 104)	43.0%	50.0%	34.0%	0.012
	Yes (*n* = 138)	57.0%	50.0%	66.0%	
Dyslipidaemia	No (*n* = 136)	56.2%			
	Yes (*n* = 106)	43.8%			

**(b):** 

	Median (P25-P75)	Depression without dyslipidaemia	Depression with dyslipidaemia	Wilcoxon test
BMI (kg/m^2^)	27.4 (24.1–32.3)	26.3 (22.6–30.4)	29.0 (26.0–34.2)	<0.001
Age (years)	44 (35–53)	42 (34–53)	47 (37–52)	0.221
Cholesterol (mg/dL)	185.5 (163.0–216.0)	183.5 (161.0–209.5)	187.5 (165.0–220.0)	0.200
HDL-C (mg/dL)	52.0 (44.0–62.0)	59.5 (52.0–69.0)	43.0 (37.0–48.0)	<0.001
Triglycerides (mg/dL)	110.0 (79.0–164.0)	86.0 (65.5–109.0)	174.5 (127.0–222.0)	<0.001
ESS	11 (7–14)	11 (7–14)	12 (8–15)	0.127
BDI	22 (18–29)	21 (17–29)	22 (18–29)	0.456
ISI	17 (14–20)	17 (14–21)	17 (13–20)	0.102
TAS-20	54 (44–63)	51 (42–63)	56 (46–63)	0.092
CRP (mg/L)	1.8 (0.7–4.4)	1.3 (0.5–4.1)	2.2 (1.1–4.7)	0.001

BMI = body mass index; OSAS = obstructive sleep apnoea syndrome; CRP = C-reactive protein; PLMs = periodic limb movements during sleep; RLS = restless legs syndrome; HDL-C = high-density lipoprotein cholesterol; ESS = Epworth Sleepiness Scale; BDI = Beck Depression Inventory; ISI = Insomnia Severity Index; TAS-20 = Toronto Alexithymia Scale.

**Table 3:** Univariate analyses (*n* = 242).

Variables	Depression without dyslipidaemia	Depression with dyslipidaemia	OR (CI 95%)	*P* value
Gender				0.201
Female	59.7%	40.3%	1	
Male	51.5%	48.5%	1.40 (0.84 to 2.34)	
BMI (kg/m^2^)				<0.001
<25	74.7%	25.3%	1	
≥25 & <30	49.4%	50.6%	3.02 (1.54 to 5.89)	
≥30	46.3%	53.7%	3.43 (1.73 to 6.77)	
Age (years)				0.206
<50	59.1%	40.9%	1	
≥50	50.6%	49.4%	1.41 (0.83 to 2.41)	
Benzodiazepine receptor agonists				0.347
No	57.6%	42.4%	1	
Yes	50.0%	50.0%	1.36 (0.72 to 2.59)	
Antidepressant therapy				0.774
No	55.6%	44.4%	1	
Yes	57.5%	42.5%	0.92 (0.54 to 1.59)	
Other psychotropic treatments				0.655
No	56.7%	43.3%	1	
Yes	52.0%	48.0%	1.21 (0.53 to 2.79)	
Smoking				0.105
No	58.8%	41.2%	1	
Yes	46.0%	54.0%	1.68 (0.90 to 3.14)	
Alcohol				0.913
No	55.0%	45.0%	1	
Occasional	57.5%	42.5%	0.90 (0.52 to 1.56)	
Regular	58.3%	41.7%	0.87 (0.36 to 2.10)	
Caffeine				0.356
No	63.6%	36.4%	1	
Yes	55.0%	45.0%	1.43 (0.67 to 3.06)	
Snoring				0.001
No	70.1%	29.9%	1	
Yes	48.4%	51.6%	2.50 (1.43 to 4.37)	
OSAS				0.001
No	65.2%	34.8%	1	
Yes	43.6%	56.4%	2.43 (1.44 to 4.11)	
Insomnia disorders				0.652
No	51.1%	48.9%	1	
Sleep deprivation alone	51.8%	48.2%	0.97 (0.37 to 2.52)	
With sleep duration ≥ 6 hours	60.2%	39.8%	0.69 (0.35 to 1.38)	
With sleep duration < 6 hours	53.2%	46.8%	0.92 (0.41 to 2.09)	
Sleep movement disorders				0.631
No	57.9%	42.1%	1	
Moderate to severe PLMs	51.6%	48.4%	1.29 (0.60 to 2.77)	
RLS alone or combined with PLMs	50.0%	50.0%	1.38 (0.62 to 3.05)	
Excessive daytime sleepiness				0.230
No	60.4%	39.6%	1	
Yes	52.7%	47.3%	1.37 (0.82 to 2.28)	
Depression severity				0.110
Mild	64.1%	35.9%	1	
Moderate	49.1%	50.9%	1.85 (1.02 to 3.36)	
Severe	59.3%	40.7%	1.23 (0.60 to 2.51)	
Hypertension				0.002
No	64.0%	36.0%	1	
Yes	43.5%	56.5%	2.31 (1.36 to 3.93)	
Type 2 diabetes				<0.001
No	62.1%	37.9%	1	
Yes	16.1%	83.9%	8.51 (3.14 to 23.07)	
CRP				0.011
<1	69.5%	30.5%	1	
≥1 & <3	47.5%	52.5%	2.52 (1.32 to 4.79)	
≥3	51.2%	48.9%	2.17 (1.14 to 4.12)	
Alexithymia				0.013
No	65.4%	34.6%	1	
Yes	49.3%	50.7%	1.94 (1.15 to 3.28)	

BMI = body mass index; OSAS = obstructive sleep apnoea syndrome; CRP = C-reactive protein; PLMs = periodic limb movements during sleep; RLS = restless legs syndrome.

**Table 4:** Multivariate analyses (*n* = 242).

Variables	Model 1, OR adjusted (CI 95%)	*P* value	Model 2, OR adjusted (CI 95%)	*P* value	Model 3, OR adjusted (CI 95%)	*P* value	Model 4, OR adjusted (CI 95%)	*P* value
Alexithymia		0.016		0.016		0.012		0.011
No	1		1		1		1	
Yes	1.94 (1.13 to 3.34)		1.96 (1.13 to 3.40)		2.11 (1.18 to 3.76)		2.15 (1.19 to 3.87)	

Model 1 = model adjusted for OSAS and snoring. Model 2 = model adjusted for OSAS, snoring, and BMI. Model 3 = model adjusted for OSAS, snoring, BMI, type 2 diabetes, and hypertension. Model 4 = model adjusted for OSAS, snoring, BMI, type 2 diabetes, hypertension, and CRP levels. BMI = body mass index; OSAS = obstructive sleep apnoea syndrome; CRP = C-reactive protein.

**Table 5:** Lipid profile according to alexithymic status (*n* = 242).

	Whole sample (*n* = 242)	Depression without alexithymia (*n* = 104)	Depression with alexithymia (*n* = 138)	*P* value
Cholesterol (mg/dL)	185.5 (163.0–16.0)	179.5 (156.0–208.0)	190.0 (166.0–226.0)	0.011
HDL-C (mg/dL)	52.0 (44.0–62.0)	52.0 (45.0–65.0)	52.0 (43.0–61.0)	0.226
Triglycerides (mg/dL)	110.0 (79.0–164.0)	99.5 (71.0–135.5)	118.5 (85.0–178.0)	0.001
	Median (P25-P75)	Median (P25-P75)	Median (P25-P75)	Wilcoxon test

HDL-C = high-density lipoprotein cholesterol.

## Data Availability

The datasets used and/or analysed during the current study are available from the corresponding author on reasonable request.

## Additional Points

*Highlights*. Dyslipidaemia is frequent (43.8%) in major depressed individuals. Alexithymia is associated with an altered lipid profile in major depression. Alexithymia is a risk factor for dyslipidaemia in major depressed individuals. Adequate management of alexithymia is essential in major depressed individuals

## Supplementary Materials

Supplementary Materials Annex 1: detailed description of self-questionnaires used. Annex 2: description of the applied polysomnography montage. Annex 3: description of scoring criteria and diagnostic criteria used for the diagnosis of sleep disorders. Annex 4: description of the confounding factors included in the univariate analysis.
