# Supplementary material for: Impact of Alexithymia on the Lipid Profile in Major Depressed Individuals
Source: J Lipids. 2022 Jun 16;2022:5450814. doi: 10.1155/2022/5450814 (PMC9225907; doi:10.1155/2022/5450814)
Supplement: Supplementary Materials — Annex 1: detailed description of self-questionnaires used. Annex 2: description of the applied polysomnography montage. Annex 3: description of scoring criteria and diagnostic criteria used for the diagnosis of sleep disorders. Annex 4: description of the confounding factors included in the univariate analysis. [file 5450814.f1.docx]

# Supplementary data

# Annex 1

Detailed description of self-questionnaires used

- The presence of depressive symptoms was investigated using Beck Depression Inventory (BDI-II). This scale consists of 21 items that may be scored from 1 to 3. The score may vary from 0 to 63. A score of 0-9 indicates no depression, 10-18 mild depression, 19-29 moderate depression, and 30-63 severe depression [1].
- Daytime sleepiness was investigated using the Epworth Sleepiness Scale. This scale consists of 8 questions that may be scored from 0 to 3 and assesses daytime sleepiness in frequent situations of daily life. The score may vary from 0 to 24. A score greater than 10 indicates excessive daytime sleepiness [2].
- The presence of insomnia symptoms was investigated using the Insomnia Severity Index. This index consists of 7 questions that may be scored from 0 to 4. The score may vary from 0 to 28. A score of 0-7 indicates no insomnia, 8-14 subclinical insomnia, 15-21 moderate insomnia, and 22-28 severe insomnia [3].

**References**

[1] Beck AT, Steer RA, Ball R, Ranieri W. Comparison of Beck Depression Inventories -IA and -II in psychiatric outpatients. J Pers Assess. 1996;67(3):588-597.

[2] Johns MW. A new method for measuring daytime sleepiness: the Epworth sleepiness scale. Sleep. 191;14(6):540-545.

[3] Morin CM. Insomnia: psychological assessment and management. Guilford Press, New York: 1993.

**Annex 2**

Description of the applied polysomnography-montage

- Two electro-oculogram channels
- Three electroencephalogram channels (Fz-Ax, Cz-Ax and Oz-Ax, where Ax was a A1A2 mastoid reference)
- One submental electromyogram channel
- Electrocardiogram
- Pressure cannula to detect the oro-nasal airflow
- Finger pulse-oximetry
- Microphone to record breathing sounds and snoring
- Plethysmographic inductive belts to measure thoracic and abdominal breathing
- Anterior tibialis electrodes

**Annex 3**

Description of scoring criteria and diagnostic criteria used for the diagnosis of sleep disorders

Obstructive apnoeas were scored if the decrease in air flow was ≥90% for at least 10 seconds whereas obstructive hypopnoeas were scored if the decrease in airflow was ≥30% for at least 10 seconds with a decrease in oxygen saturation of 3% or followed by microarousal [1]. The obstructive apnoea-hypopnoea index correspond to the total number of obstructive apnoeas and hypopnoeas divided by the period of sleep in hours. Obstructive sleep apnoea syndrome was considered as present when the obstructive apnoea-hypopnoea index was ≥5/hour [2].

Periodic limb movements during sleep were scored on the basis of the following strict criteria: 1) duration between 0.5 to 10 seconds, 2) interval between 5 and 90 seconds from leg movement onset and 3) movements had to be part of a series of ≥4 consecutive movements meeting these criteria [3]. Periodic limb movement’s index corresponds to the total number of periodic limb movements during sleep divided by period of sleep in hours. Moderate to severe periodic limb movements during sleep were considered to be present when the periodic limb movement’s index was ≥15/hour [4]. Moreover, the diagnoses of restless legs syndrome were made according to the diagnostic criteria of the *International Restless Legs Syndrome Study Group* [5].

Finally, potential diagnoses of insomnia disorders were made according to the diagnostic criteria of the *American Academy of Sleep Medicine Work Group* [6] whereas short sleep duration was defined as sleep time <6 hours [7].

**References**

[1] Berry RB, Budhiraja R, Gottlieb DJ, Gozal D, Iber C, Kapur VK, Marcus CL, Mehra R, Parthasarathy S, Quan SF, Redline S, Strohl KP, Davidson Ward SL, Tangredi MM; American Academy of Sleep Medicine. Rules for scoring respiratory events in sleep: update of the 2007 AASM Manual for the Scoring of Sleep and Associated Events. Deliberations of the Sleep Apnea Definitions Task Force of the American Academy of Sleep Medicine. J Clin Sleep Med. 2012;8(5):597-619.

# [2] Fleetham J, Ayas N, Bradley D, Ferguson K, Fitzpatrick M, George C, Hanly P, Hill F, Kimoff J, Kryger M, Morrison D, Series F, Tsai W; CTS Sleep Disordered Breathing Committee. Canadian Thoracic Society guidelines: diagnosis and treatment of sleep disordered breathing in adults. Can Respir J. 2006;13(7):387-392.

# [3] Ferri R, Koo BB, Picchietti DL, Fulda S. Periodic leg movements during sleep: phenotype, neurophysiology, and clinical significance. Sleep Med. 2017;31:29-38.

# [4] Haba-Rubio J, Marti-Soler H, Tobback N, Andries D, Marques-Vidal P, Vollenweider P, Preisig M, Heinzer R. Clinical significance of periodic limb movements during sleep: the HypnoLaus study. Sleep Med. 2018;41:45-50.

# [5] Allen RP, Picchietti DL, Garcia-Borreguero D, Ondo WG, Walters AS, Winkelman JW, Zucconi M, Ferri R, Trenkwalder C, Lee HB; International Restless Legs Syndrome Study Group. Restless legs syndrome/Willis-Ekbom disease diagnostic criteria: updated International Restless Legs Syndrome Study Group (IRLSSG) consensus criteria--history, rationale, description, and significance. Sleep Med. 2014;15(8):860-873.

[6] Edinger JD, Bonnet MH, Bootzin RR, Doghramji K, Dorsey CM, Espie CA, Jamieson AO, McCall WV, Morin CM, Stepanski EJ; American Academy of Sleep Medicine Work Group. Derivation of research diagnostic criteria for insomnia: report of an American Academy of Sleep Medicine Work Group. Sleep. 2004;27(8):1567-1596.

[7] Hein M, Lanquart JP, Loas G, Hubain P, Linkowski P. Insomnia with short sleep duration as risk factor for type 2 diabetes: a systematic review of the literature. Rev Med Brux. 2020;41(2):98-104.

**Annex 4**

Description of the confounding factors included in the univariate analysis

After a review of the literature on the potential factors associated with dyslipidaemia [1-9], the potential confounding factors included in this study were body mass index (categorised: <25 kg/m², ≥25 & <30 kg/m², ≥30 kg/m²), age (categorised: <50 years, ≥50 years), alcohol consumption (categorised: no, occasional, regular), insomnia disorders (categorised: no, sleep deprivation alone, wit sleep duration ≥6 hours, with sleep duration <6 hours), sleep movement disorders (categorised: no, moderate to severe periodic limb movements during sleep alone, restless leg syndrome alone or combined with periodic limb movements during sleep), depression severity (categorised: mild, moderate, severe), CRP levels (categorised: <1 mg/L, ≥1 & <3 mg/L, ≥3 mg/L) and as binary variables: gender, benzodiazepine receptor agonists, antidepressant therapy, other psychotropic medications (antipsychotics or thymostabilisers), smoking, caffeine consumption, snoring, obstructive sleep apnoea syndrome, excessive daytime sleepiness, hypertension and type 2 diabetes.

**References**

[1] van Reedt Dortland AK, Giltay EJ, van Veen T, Zitman FG, Penninx BW. Longitudinal relationship of depressive and anxiety symptoms with dyslipidemia and abdominal obesity. Psychosom Med. 2013;75(1):83-89.

[2] Onat A, Can G, Hergenç G. Serum C-reactive protein is an independent risk factor predicting cardiometabolic risk. Metabolism. 2008;57(2):207-214.

[3] Karr S. Epidemiology and management of hyperlipidemia. Am J Manag Care. 2017;23(9 Suppl):S139-S148.

[4] Zhan Y, Zhang F, Lu L, Wang J, Sun Y, Ding R, Hu D, Yu J. Prevalence of dyslipidemia and its association with insomnia in a community based population in China. BMC Public Health. 2014;14:1050.

[5] Drager LF, Santos RB, Silva WA, Parise BK, Giatti S, Aielo AN, Souza SP, Furlan SF, Lorenzi-Filho G, Lotufo PA, Bensenor IM. OSA, Short Sleep Duration, and Their Interactions With Sleepiness and Cardiometabolic Risk Factors in Adults: The ELSA-Brasil Study. Chest. 2019;155(6):1190-1198.

[6] Innes KE, Selfe TK, Agarwal P. Restless legs syndrome and conditions associated with metabolic dysregulation, sympathoadrenal dysfunction, and cardiovascular disease risk: a systematic review. Sleep Med Rev. 2012;16(4):309-339.

[7] Shen Z, Munker S, Wang C, Xu L, Ye H, Chen H, Xu G, Zhang H, Chen L, Yu C, Li Y. Association between alcohol intake, overweight, and serum lipid levels and the risk analysis associated with the development of dyslipidemia. J Clin Lipidol. 2014;8(3):273-278.

[8] Delacrétaz A, Vandenberghe F, Gholam-Rezaee M, Saigi Morgui N, Glatard A, Thonney J, Solida-Tozzi A, Kolly S, Gallo SF, Baumann P, Berney S, Zulauff SV, Aubry JM, Hasler R, Ebbing K, von Gunten A, Conus P, Eap CB. Early changes of blood lipid levels during psychotropic drug treatment as predictors of long-term lipid changes and of new onset dyslipidemia. J Clin Lipidol. 2018;12(1):219-229.

[9] Grosso G, Marventano S, Galvano F, Pajak A, Mistretta A. Factors associated with metabolic syndrome in a mediterranean population: role of caffeinated beverages. J Epidemiol. 2014;24(4):327-33.
